# An unusual case of systemic amyloid causing constrictive heart failure

**DOI:** 10.1007/s12471-016-0890-y

**Published:** 2016-08-18

**Authors:** F. A. A. Mohamed Hoesein, M. J. Swaans, L. S. Jiwa, C. A. Seldenrijk, H. W. van Es

**Affiliations:** 1 Department of Radiology, St. Antonius Hospital Nieuwegein, Nieuwegein, The Netherlands; 2 Department of Cardiology, St. Antonius Hospital Nieuwegein, Nieuwegein, The Netherlands; 3 Pathology and DNA, St. Antonius Hospital Nieuwegein, Nieuwegein, The Netherlands

A 74-year-old male was analysed for exertional dyspnoea in our outpatient clinic. Cardiac MRI showed diffuse enhancing pericardial soft-tissue (Fig. 1a), but no late gadolinium myocardial enhancement. PET-CT showed the pericardial and peri-renal soft-tissue mass to be calcified, however without FDG uptake. Surgical biopsy of the omentum revealed amyloid depositions fitting with a diagnosis of systemic amyloid. Systemic therapy was started, but the heart failure was progressive and not responding to medical therapy. On repeat cardiac MRI flattening of the ventricular septum during expiration was seen during free breathing (online movie 1). Postmortem macroscopic examinations of the heart and kidney showed extensive peri-renal and pericardial amyloid deposition (Fig. 1b).

**Fig. 1:**
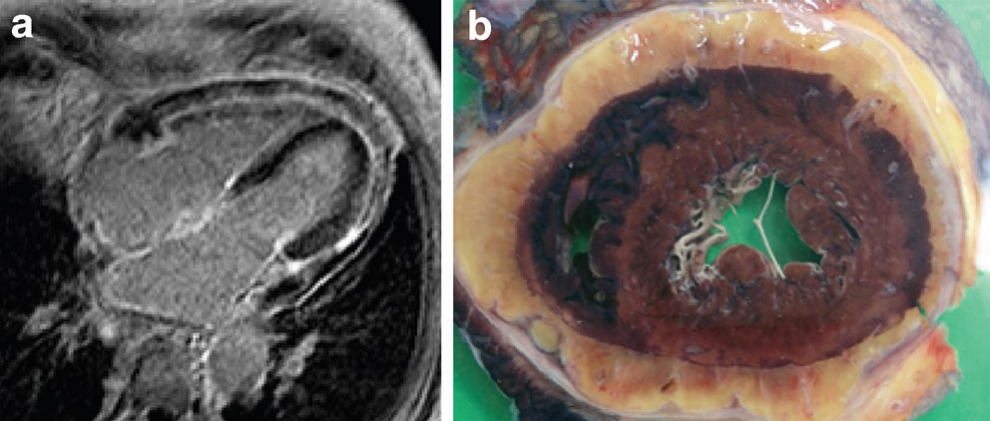
**a** Patchy enhancing pericardial soft-tissue on late-gadolinium enhanced MRI, **b** Macroscopic image of the heart with pericardial amyloid deposition

Cardiac amyloid is a rare systemic disease (6 per million cases). In most cases there is restrictive heart failure because of amyloid depositions. The usual pattern seen on cardiac MRI is late gadolinium enhancement of the entire subendocardial ring. It is very rare for systemic amyloid to present with pericardial amyloid depositions causing severe constrictive heart failure [1–3].

## Caption Electronic Supplementary Material

Movie: Real-time cine image short-axis showing flattening and paradoxical movement of the interventricular septum which is a sign of constrictive pericarditis.
